# Mindful organizing in patients' contributions to primary care medication safety

**DOI:** 10.1111/hex.12689

**Published:** 2018-04-14

**Authors:** Denham L. Phipps, Sally Giles, Penny J. Lewis, Kate S. Marsden, Ndeshi Salema, Mark Jeffries, Anthony J. Avery, Darren M. Ashcroft

**Affiliations:** ^1^ NIHR Greater Manchester Patient Safety Translational Research Centre Manchester Academic Health Science Centre (MAHSC) The University of Manchester Manchester UK; ^2^ Division of Pharmacy and Optometry School of Health Sciences The University of Manchester Manchester UK; ^3^ Division of Population Health Health Services Research and Primary Care School of Health Sciences The University of Manchester Manchester UK; ^4^ Division of Primary Care School of Medicine The University of Nottingham Queens' Medical Centre Nottingham UK

**Keywords:** high‐reliability organizing, medication safety, mindful organizing, organizational safety, patient safety, primary care

## Abstract

**Background:**

There is a need to ensure that the risks associated with medication usage in primary health care are controlled. To maintain an understanding of the risks, health‐care organizations may engage in a process known as “mindful organizing.” While this is typically conceived of as involving organizational members, it may in the health‐care context also include patients. Our study aimed to examine ways in which patients might contribute to mindful organizing with respect to primary care medication safety.

**Method:**

Qualitative focus groups and interviews were carried out with 126 members of the public in North West England and the East Midlands. Participants were taking medicines for a long‐term health condition, were taking several medicines, had previously encountered problems with their medication or were caring for another person in any of these categories. Participants described their experiences of dealing with medication‐related concerns. The transcripts were analysed using a thematic method.

**Results:**

We identified 4 themes to explain patient behaviour associated with mindful organizing: knowledge about clinical or system issues; artefacts that facilitate control of medication risks; communication with health‐care professionals; and the relationship between patients and the health‐care system (in particular, mutual trust).

**Conclusions:**

Mindful organizing is potentially useful for framing patient involvement in safety, although there are some conceptual and practical issues to be addressed before it can be fully exploited in this setting. We have identified factors that influence (and are strengthened by) patients’ engagement in mindful organizing, and as such would be a useful focus of efforts to support patient involvement.

## 1 BACKGROUND

Medicines can prevent or treat many conditions and so are the most common intervention in health care, with over 1 billion prescription items being issued in English primary care each year.^1^ The increasing prevalence of long‐term or multiple health conditions managed in the community^2^ and a trend towards greater integration between primary and secondary care services^3^ mean that primary care makes an important contribution to population health. It also means, though, that medicines management in this setting is increasingly high‐risk, with hazards including the supply of inappropriate medication and insufficient monitoring of medicines usage.^4^ Epidemiological data from the United Kingdom suggest a prevalence of approximately 5% for potentially hazardous prescribing and between 7% and 11% for omitted monitoring,^5^, ^6^ while a systematic review^7^ estimated a median prevalence of 13% for harmful adverse drug events in North American, European and Australian ambulatory care. Hence, there is benefit in identifying ways of improving primary care medication safety. Amongst the issues that have been explored is the role of patients, their carers and their representatives in ensuring safe practice, but while there is a prima facie argument for involving health‐care service users, it remains unclear how—and to what extent—they can make an effective contribution to patient safety.^8^ One source of insights about patient safety, which might be addressed to the issue outlined here, is the research on safety across different work settings.^9^ In this study, we examine how one theory that has been applied to organizational safety can be used to understand patient involvement in medication safety.

### 1.1 Mindful organizing and patient safety

High‐reliability theory^10^, ^11^ attempts to characterize what organizations that avoid failure in high‐risk activities do to maintain reliability. According to Weick & Sutcliffe,^11^ so‐called high‐reliability organizations (HROs) demonstrate particular characteristics in the way they operate: anticipating problems (being aware of what is happening in the work system; being alert to ways in which an incident could occur; looking beyond simplistic explanations for incidents); and containing problems (being prepared to deal with contingencies; using relevant expertise regardless of where it is situated within the organizational hierarchy). Vogus & Sutcliffe^12^ proposed “mindful organizing” as a collective mental orientation in which the organization continually engages with its environment, reorganizing its structures and activities as necessary, rather than mindlessly executing plans in ignorance of the prevailing circumstances. This is a dynamic social process, consisting of specific actions and interactions between those engaged in frontline organizational work. It creates the context for thought and behaviour across the organization, but is relatively transient and so needs to be actively maintained.^11^

The extent to which high‐reliability theory applies to organizational safety in general, and patient safety in particular, has been the subject of some debate.^13^, ^14^ Leveson et al^15^ noted that high‐reliability theory was based on a specific type of organization—one in which the work system is relatively stable and its characteristics well understood—and argue that it is not generalizable to others. They further argue that reliability and safety are not necessarily equivalent or even compatible properties of a work system; therefore, high‐reliability theory is less applicable to *safety* than has been assumed. However, Hollnagel^16^, ^17^ and Sujan et al^18^ conceive of safety in terms of resilience—an organization's capacity to maintain successful work in the face of varying conditions. Hollnagel attributes resilience to an organization's mindfulness (in Weick's sense of the term), thus implying a link between high‐reliability theory and organizational safety. Similarly, Hopkins^19^ argues that the characteristics of a HRO and the components of safety culture suggested by Reason^20^ (reporting; flexibility; learning; fairness) are broadly equivalent. As for empirical data, Roberts et al^21^ demonstrated the benefit for patient outcomes of a paediatric intensive care unit adopting high‐reliability principles, while Vogus & Sutcliffe^22^ examined the relationship between mindful organizing and patient safety using a survey of nurses in the United States. The latter study found a negative relationship between the level of mindful organizing and the number of reported medication errors and patient falls.

While there appears to be convergence between the various concepts described here (i.e. high‐reliability organizing, resilience and safety culture), a particular insight offered by the literature on mindful organizing is to emphasize its grounding in social relations.^23^, ^24^, ^25^ In other words, the collective capacity to understand, anticipate and respond to problems both depends on and subsequently provides a structure for social interactions such as collaboration and negotiation.^23^, ^26^ The potential relevance of this insight to patient safety is demonstrated by examining the issue of patient involvement.

### 1.2 Patients’ contributions to medication safety

Previous research suggests that patients could be involved in the prevention of safety issues.^27^, ^28^, ^29^, ^30^ Roles for patients include reporting adverse events, notifying or questioning health‐care professionals in the case of any concerns, and providing relevant information about their medicines or health conditions.^31^, ^32^, ^33^, ^34^, ^35^ Yet, involving patients in safety is not necessarily a straightforward matter. First, involvement occurs in the context of a relationship between patients and health‐care professionals, such that patients feel more inclined to involve themselves when they perceive that they will be treated with respect and their contributions heard and taken seriously.^36^, ^37^, ^38^ Indeed, a study of patients’ perceptions about threats to safety^39^ found that a breakdown in the relationship between patient and clinician was a more prevalent concern than was a technical error such as an adverse drug event, despite the latter typically being the main concern of health‐care professionals. A second issue is that patients’ involvement is informed by their understanding of the problem at hand. Patients vary in their belief that safety is a priority in their care, or even a distinct issue (as opposed to being an assumed part of their care); they also draw upon accumulated knowledge and experience about their care in deciding whether and how to act.^37^, ^40^, ^41^ Third, patients will be more inclined to become involved in safety activities if they perceive that they have the capacity and means to do so and that doing so will have a positive effect.^8^, ^37^, ^41^

From a mindful organizing perspective, patient involvement might be conceptualized as a set of interactions between patients and health‐care professionals that maintain collective “mindfulness” about safety issues, that is an awareness of potential or impending patient safety hazards and a capacity for acting on such insights.^14^ Through these interactions, patients and their families or carers can contribute to mindfulness by amplifying otherwise weak signals of patient safety problems, questioning issues that would otherwise be taken for granted, and raising concerns that would otherwise be missed. Table 1 describes some examples of potential contributions that follow from the elements of mindful organizing listed by Weick et al.^11^, ^42^

**Table 1:** Examples of patients’ contribution to mindful organizing

Element	Definition	Example of patient contribution
Preoccupation with failure	Being constantly aware of the potential for an unexpected event that could compromise patient safety	Querying the substitution of prescribed medication by a pharmacist
Reluctance to simplify medication interpretations	Questioning assumptions and received wisdom to create a more complete and nuanced understanding of risks	Cross‐checking different sources of advice (eg doctor; medicines literature)
Sensitivity to operations	On‐going interaction and information sharing about human and organizational factors that are influencing the current safety level	Highlighting specific communication needs and suggesting ways of providing for these needs
Commitment to resilience	Maintaining a capability to detect, contain, recover and learn from an adverse event before it causes further harm	Identifying a medication error and discussing the error with health‐care professionals
Deference to expertise	When dealing with a problem, allowing decisions to be made by those with the most expertise, regardless of formal role	Where capable, engaging in shared decision making about medication usage

Definitions adapted from Vogus & Sutcliffe.^12^

Given its apparent relevance to patient safety, our study aimed to examine ways in which patients might contribute to mindful organizing. To do so, we drew from primary care patients’ experiences of dealing with medication safety issues.

## 2 METHOD

### 2.1 Design and sampling

The study used a qualitative design. The sampling frame was members of the public in North West England and the East Midlands who either had a long‐term health condition requiring medicines usage, were taking several medicines, had previous experience of problems with their medicines or were carers of people in any of these groups. This frame was chosen on the basis of evidence that patients with long‐term conditions or on multiple medications are at increased risk of medication‐related problems.^43^ We identified members of the frame in 3 ways: (i) a list of people who had previously expressed interest in patient safety research at our institution; (ii) radio, print and social media advertisements; and (iii) local community organizations who assisted with recruitment (e.g. patient advocacy and cultural groups). Recruitment was on an opt‐in basis, with participants receiving a gift voucher and travel expenses in return for taking part. Ethical approval for the study was obtained from the National Health Service Research Ethics Committee (reference 13/LO/1531) and The University of Nottingham Medical School Ethics Committee.

### 2.2 Data collection

Data were collected primarily through focus groups. The size of each group ranged from 3 to 11 participants, depending on participant availability. One‐to‐one interviews were conducted with participants who were unable to attend a focus group. Each focus group or interview was led by one member of the research team (DLP, SG, PJL, KM or NS), with another member of the research team, or a layperson from our research group's public and patient involvement panel, acting as a cofacilitator. Three focus groups were conducted for participants who did not speak English as a first language: 1 in Urdu; 1 in Hindi; and 1 in British Sign Language (BSL). For the Urdu and Hindi groups, a researcher who was fluent in the respective language acted as the lead facilitator, with a member of the research team as cofacilitator. The third group was facilitated by DLP with a BSL interpreter external to the research team acting as cofacilitator.

A semi‐structured topic guide was used to guide each discussion. This included the following topics: problems that participants had experienced with medicines; their interactions with doctors and pharmacists; their own contribution to safe medication use; their knowledge of medication reviews; and adverse event reporting. Each session lasted for between 75 and 120 minutes and, with the consent of all participants, was audio‐recorded and transcribed.

### 2.3 Data analysis

The transcripts were analysed using an inductive thematic approach.^44^ Initially, the focus of the analysis was on instances of patients being involved, or attempting to be involved, in patient safety activities. Four members of the research team (DLP, SG and PJL and KM) separately reviewed the same 3 transcripts within the data set to identify emerging themes related to patient involvement. The research team members then discussed and agreed on a set of themes that appeared to distinguish between the successful and unsuccessful attempts at involvement described by participants. These themes were subsequently applied across the whole data set by 3 of the researchers (DLP, SG and PJL). When comparing the participants’ accounts, the first author, who was familiar with safety science research, noted that the accounts varied in the extent to which they demonstrated the elements of mindful organizing listed in Table 1. Therefore, literature on this topic was used to inform interpretation of the themes. Finally, the findings were reviewed by the other members of the research team (TA, DA, MJ and NS) to ensure that they adequately reflected both the content of the transcripts and the research question. Version 10 of NVivo was used to document the analysis.

## 3 FINDINGS

A total of 126 participants took part in a focus group or interview. Table 2 shows the type and number of patients in each session. We identified 4 themes that explained patients’ contributions to mindful organizing: knowledge; communication; artefacts; and relationships.

**Table 2:** Participants in the study

Participant type	N
Focus groups	
Generic patient group	
Session 1	11
Session 2	7
Session 3	11
Session 4	9
Parents of children with a long‐term condition	4
Renal patients	8
Cardiovascular patients	
Session 1	10
Session 2	9
Mental health service users	
Session 1	3
Session 2	7
People recovering from substance misuse	6
Members of a male‐to‐female transgender group	3
Members of a Deaf group (BSL speakers)	6
Members of a visually impaired group	3
Elders	
White (Session 1)	3
White (Session 2)	3
Black Afro‐Caribbean	9
Asian (Urdu speakers)	8
Asian (Hindi speakers)	4
Interviews	
Carer of a mental health patient	1
Visually impaired service user	1

### 3.1 Knowledge

Participants’ accounts referred to their assimilating and using knowledge, either about the clinical indication for their medication use or about the system within which their medicines were supplied. Some participants—typically those with long‐term or complex medication needs—described having learned about the need to ensure that medication safety risks are properly managed during their interactions with health‐care organizations.

[I have] an allergy to Penicillin, so [I have] to look for that word on [the label] to make sure it's not inside. [But] in an emergency, if I was to go to the hospital [for example, the staff] don't know that I've got this allergy, it's not on their records, I have to tell them directly [about it] so they don't give [Penicillin to] me. [Deaf group]

On a number of occasions I've had to be careful that [the pharmacist doesn't] give [me] something that's the nearest substitute [for a branded product]. […] When I was on ciclosporin [and tacrolimus], [the pharmacist said] […] “we can't get [those], [instead] we're going to get you [generics]” […] [I said] “no you're not.” […] The dosage needs to be changed with the different type, and […] I know of cases where patients have become seriously ill because of that. [Renal conditions group]

The insights of these “expert patients,” gained from personal or vicarious experience, seemed to provide them with a basis for playing a proactive role in medication safety. With regard to mindful organizing, both demonstrate a preoccupation with failure, being concerned to ensure that they do not receive inappropriate medication. In addition, the second participant describes a reluctance to simplify interpretations, in that he draws the pharmacists’ attention to a source of risk they may otherwise overlook. However, being an experienced medication user was not always a sufficient safeguard against medication‐related problems. One participant describes how, despite taking precautions with her medication usage, she was affected by a medication hazard of which she was unaware.

My psychiatrist was on holiday, so I [saw] this locum and he [suggested] changing my medication. I [said] okay. I'd also gone to the [GP] because I was ill, and they [gave] me antibiotics. I knew that because I'd taken antibiotics I can't take the cholesterol tablets and I have to lower the dose of my warfarin. But what nobody told me was the antibiotics and the tablets for my mental health, you can't take them together because the effect of it may make you absolutely [incapacitated]. It was awful. [Mental health group]

While this participant also demonstrates a preoccupation with failure, its effect is constrained by her lack of knowledge about one source of failure (the unrecognized interaction hazard). Recognizing and attempting to mitigate the fragmented nature of the prescribing—carried out by different health‐care professionals in different locations, possibly with a limited understanding of the *overall* clinical picture—may have also led to an awareness of the additional hazard, thus demonstrating the effect of sensitivity to operations. These observations suggest that, similarly to the argument cited earlier,^23^, ^26^ participants’ knowledge both informs and is informed by mindful organizing.

### 3.2 Communication

The experience of the mental health patient highlights the interactive nature of mindful organizing; in that instance, the patient's work in preventing an adverse event assumed that the health‐care professionals recognized and informed her about relevant sources of risk. In our study, communication between patients and health‐care professionals was often mentioned in relation to patients’ involvement in medication safety activities.

When I asked [the pharmacist] what these tablets were they took me into a room and told me everything I needed to know. Because I kept [asking the doctor] “why am I on three [different] blood pressure tablets?” They were for different things. [Long‐term conditions group]

I phoned [the practice] and said, I'm [already] on this medication, and [was then prescribed] some vitamin A, [but] according to the [information leaflet I] mustn't take it. [The receptionist] said, “well just don't take it [and] don't worry about it then,” which is [the GP's] normal answer anyway. [Visually impaired group]

These accounts differ in the degree to which the communication led to an improvement in knowledge about the patient's medication. In the first instance, the pharmacist responded to the patient by helping to educate him about his medication. In the second, the patient was alerted to a potential risk by the information leaflet, but he gained no further knowledge from his attempt to discuss it—and it is not clear whether his GP gained any further insight about the patient's medication needs either. What both accounts do have in common is that they illustrate patients engaging in mindful organizing (in both cases, demonstrating a reluctance to simplify interpretations) in an attempt to compensate for knowledge gaps left by the health‐care professionals.

### 3.3 Artefacts

The physical artefacts involved in medicines management provide further ways to support mindful organizing. As suggested in the previous example, medication labels and information leaflets provided a standardized source of knowledge about medicines, which alerted several of the participants to potential medication risks. In doing so, they facilitated a preoccupation with failure and a reluctance to simplify operations.

I said to the GP, “am I allowed to take [these tablets], because […] I've had a transplant?” He went, “oh yes, you can take them.” I got them home and […] read the leaflet [which] said, do not take. So, I [rang] the hospital, [who] said [definitely] do not take them. [Renal conditions group]

However, it was evident from the data that medication labels and leaflets were used inconsistently across the sample. One barrier that affected some of the participants was a lack of accessibility, due to either the format (in the case of the visually impaired group) or language difficulties (in the case of the hearing‐impaired and Asian elder groups). From a mindful organizing perspective, these problems highlight the value of sensitivity to operations, which would facilitate patients and health‐care professionals to collaborate in addressing the communication needs of the former.

If you are blind or partially sighted how do you read your medication label if you don't read braille? Because a lot of people assume that the braille on packaging is the detail, but it isn't, it's the product. So when the pharmacist puts a sticky label on the packet for the individual, if you can't see that label, which is virtually anybody who is sight impaired, then actually how are they going to be compliant? [Visually impaired group]

For those who were more easily able to access information leaflets, a further barrier was finding them to be uninformative. This too might be understood as a challenge to mindful organizing, for which patients might compensate through a reluctance to simplify operations.

You can't actually rely on those information leaflets in the box because sometimes there is stuff that has been updated or different manufacturers have done different tests. […] I looked at the […] internet and found extra information from […] either the manufacturer or things like the NHS website. So that it's more than just reading the print out that you get at the time. [Renal conditions group]

Other aspects of physical design—for example colour—may also serve to inform patients about their medication and help them to identify any anomalies. However, participants’ accounts suggest that, as with labels and leaflets, physical features vary in their informational value. For example, some patients, but not others, are able to use colour as a reliable guide to identifying their medication.

I checked my insulin when I got it and [noticed that] they had written on the [container] the right [name]—Levemir, which is [what] I take—[but] it had like an orange thing on it and I thought, oh that's [unusual]. […] It [turned out to be] the right make […] but it was the wrong strength. [Cardiovascular conditions group]

Because of the medication I'm on, if I take antibiotics I'm not allowed to take the cholesterol tablets, but I just have to guess I'm throwing away the right one, because it used to be orange, and now they're white. And I take other white tablets of an evening. [Mental health group]

Both accounts illustrate how a commitment to resilience may be present in, or absent from, the design and supply of medicinal products. Furthermore in the second example, the mindlessness that is represented in the medication's colour‐coding is compounded by an apparent lack of compensatory mindfulness on the part of the patient, who might otherwise demonstrate a sensitivity to operations by raising the problem with those involved in medication supply.

### 3.4 Relationships and trust

Implicit to all of the participants’ accounts is a common theme: their trust in the health‐care system. There are a number of ways in which trust appears to operate. In some cases, the participants suggested a relatively high level of trust in the system to work as they expected (eg when assuming that health‐care professionals would detect and communicate all prescribing hazards). In others, the participants suggested a relatively low level of trust (eg when doubting how seriously one's concerns about medication are being taken by the GP). The participant's trust could be appropriately placed (eg if medicines information sought from a GP or pharmacist is correct) or inappropriately placed (eg if medication is misidentified). Some participants referred to the use of “trusted” collaborators or aids, which varied in terms of their formality and their degree of improvisation.

I went into [the pharmacy] and said, “I can't read my medication label, I believe that there is such a thing as a talking label.” [Fortunately] I got a really proactive pharmacist [who went to] find out about it. [So] they stick the printed label on as usual, and then the pharmacist goes out the back and verbally dictates what's on the label. [Visually impaired group]

[I was once prescribed] Penicillin, even though I am allergic to [it]. [My daughter‐in‐law] saw it and said “you can't take that.” I always let my daughter‐in‐law check the medicine first. She said that if she hadn't checked [that one], then I would have had to be taken to the hospital. [Asian elders group]

In principle, the measures adopted by these participants serve to mitigate the risks associated with medication usage. From a mindful organizing point of view, they result from sensitivity to operations involving the participants and they contribute to resilience in the patients’ medication usage. However, they may not always offer as reliable a risk control as those involved assume. For example, in the second example, the safety of the arrangement depends on the mediator's ability to detect any hazards that are present.

While the foregoing examples highlight the patient's level and focus of trust, another variable in patient interactions with health‐care professionals is the degree of trust that the health‐care professionals have in patients. As the following excerpts show, perceived trust on the part of health‐care professionals can be as complex a matter as it is on the part of the patient; with regard to mindfulness, they suggest that expertise in identifying and dealing with a particular medication issue, as well as a preoccupation with failure, may reside with either or both parties.

I went to [the GP] and I said, I think [my medicines are] contradicting with each other. […] He said, no, I'm the doctor, I know about medication, nothing is contradicting, it's all in your head. […] [Two years later, another GP noticed that this participant had a high blood pressure]. […] 226 over 126. He checked it six times. Going through my medication […] he says, do you know what? This, this and this […] contradict with each other. [Mental health conditions group]

I get my bloods done every 13 weeks. [When the result] comes back it tells not only me but [also my doctor] how my medication is doing. [If the results are outside the expected range] my doctor tends then to ask me do I want to [adjust] my medication. […] [So] the onus is not just on the doctor […], it's on yourself as well. [Transgender group]

GPs and pharmacists [might have to ask], does this [patient] genuinely know [about their medicines] or are they flannelling it? Are they pretending to know? Because that can be as dangerous, can't it? [Visually impaired interview]

## 4 DISCUSSION

Our findings illustrate that patients could potentially contribute to the “mindfulness” of medicines management. This potential is realized through 4 interacting processes: assimilating and applying knowledge about medication risks; communicating with health‐care professionals; using artefacts; and recognizing the level of trust that can be placed in each of the parties involved. Conversely, a weakness or absence of these processes will limit the contribution that a patient can make. Also, while these processes contribute to mindful organizing, they are in turn informed and shaped by the mindful organizing that occurs.

Given that mindful organizing is considered to be grounded in social interactions and serves to improve understanding of on‐going risks, it would appear to lend itself to the examination of patient involvement in safety. However, some conceptual issues arise in relation to our study findings. First, as described in the previous section, trust can operate in various ways. In general terms—at least, with regard to health‐care professionals and their own managers—there appears to be a positive relationship between trust and mindful organizing,^22^ and a high level of trust has been found to enhance the relationship between mindful organizing and medication error rates.^45^ A further consideration, though, is the argument by Entwistle & Quick^46^ that a patient may place trust in a health‐care professional having considered, and remaining vigilant to, patient safety risks (ie mindfully); alternatively, the patient may do so in ignorance of any such risks (ie mindlessly). Entwistle & Quick also use the example of a patient challenging a health‐care professional to illustrate that a given behaviour could be indicative of high trust (eg in the belief that the 2 are working together to anticipate any patient safety risks) or low trust (eg in the belief that low competence on the part of the health‐care professional will be exposed). It might be surmised that a health‐care professional reacts differently to a patient's challenge based on the perceived level of trust that the patient is demonstrating, or that there might be individual differences between clinicians regarding their openness to being questioned, hence an apparent attempt at mindful organizing having different outcomes. There would appear to be merit in examining in more detail how trust and mindful organizing influence each other, for example by examining how levels and targets of trust change as an organization becomes more or less mindful, as in Roberts et al's case study.^21^

A second issue is the intentionality of mindful organizing. Levinthal & Rerup^47^ depict mindful behaviour as being conscious and effortful, as opposed to less mindful behaviour which is automatic and routinized; if this depiction holds, as Vogus & Sutcliffe^22^ argue that it does, then a question arises as to what was intended by the patient behaviours described in the current study. It would seem that many of the behaviours were intended to help ensure safe medication usage; possibly they were knowingly motivated by particular aspects of mindful organizing (eg a concern about potential failures). Whether they were explicitly intended to achieve mindful organizing per se, though, is less clear; none of the participants stated this to be the case. How important, then, is it for patients and health‐care providers to engage in mindful organizing in the sense that we have defined it here? We would argue that this concept provides a way of understanding what patients *could* do to contribute to patient safety, with support from health‐care providers as necessary, as opposed to what they are currently observed to do. Alternatively, echoing the argument made by Hopkins,^48^ it provides a model against which observed patient involvement in safety can potentially be compared. However, we also note the argument made previously^47^ that while mindful organizing needs conscious effort, its role may be to interact with, rather than completely replace, less mindful routinized behaviour that presumably can be sustained with less effort on a day‐to‐day basis. In fact, if mindful organizing is held to be effortful, excessive reliance on health‐care professionals to provide it may be undesirable or even counterproductive given the burdens already imposed by their work.^49^

Our findings provide additional insight into the circumstances under which patient involvement occurs. Applying the notion of mindful organizing highlights the importance of considering how patients’ actions interact with other parts (human or artefact) of the medicines management system. In other words, the extent to which patient action or inaction contributes to medication safety depends on the extent to which it complements other risk controls present in the system. Mindful organizing on the part of patients can compensate to a degree for a lack of mindful organizing on the part of health‐care providers and therefore is beneficial in its own right. However, a lack of reciprocation of, or support for, a patient's efforts in mindful organizing could lead to them being undermined or thwarted, possibly without either party realizing this is the case. Alternatively, these efforts may be successful in mitigating risks but, if the patient's work is unseen, the risks themselves may remain obscured too; hence, the system is assumed to be safer than it actually is. Therefore, patient involvement should not be treated simply as an independent safety intervention, and nor should it be assumed or expected to occur of its own accord; rather, it should be treated as a deliberate strategy to be integrated with other safety‐related activities within the medicines management system, as well as depending on the interest and ability of individual patients.^34^, ^50^

A mindful organizing approach to involving patients in safety might, though, raise some issues in implementation. As mindful organizing serves to amplify signals of potential risks, one issue concerns the need for a way to distinguish those signals that accurately point to risks from those that are irrelevant.^23^ Another issue concerns the foundation of mindful organizing on tight social coupling around a set of core values.^42^ Previous studies have noted that patient safety is not objective but contingent and negotiable between the different parties involved.^37^, ^40^ In which case, how are the core values regarding patient safety agreed between patients and health‐care professionals? It is difficult to provide any definitive answers to these issues on the basis of our current data. We surmise that they might be resolved between patients and health‐care professionals—at least in part—on the basis of the factors identified in this study (eg as they develop mutual knowledge about risks and trust in each other's judgement). At a broader level, a mindful organizing approach may be supported by particular norms and practices within health‐care organizations, for example, ones that foster knowledge, information sharing, divergent thinking and a repertoire of skills to deal with various situations.^21^, ^42^, ^51^ Similar suggestions have been made from a resilience perspective^52^, ^53^, ^54^; particularly relevant to the current study, Schubert et al.^54^ discuss the importance of incorporating patients and families into the pool of distributed and co‐operative expertise that is applied to patient care. In practical terms, we suggest that mindful organizing would be supported by, amongst other things, an agreed set of expectations between health‐care professionals and patients, and by the provision of tools, processes and infrastructure to support patients in understanding and communicating about safety issues. The working relationship is likely to be one that is founded on mutual trust but that allows the parties involved to question and modify their collective work as necessary. Incidentally, returning to Levinthal and Rerup's previously cited observation, it might be the case that artefacts of the kind mentioned in our study data (e.g. information repositories or medication design features) could serve to aid the work of mindful organizing.

With regard to methodology, the current study builds upon previous research on patient involvement in medication safety by focusing on primary care; it also includes insights from a large and diverse sample of service users, including participants from traditionally “underrepresented” demographic groups. The use of focus groups and interviews allowed for in‐depth exploration of the issues raised by participants. However, the study is limited by the nature of the sampling and data collection, which meant that we were unable to corroborate participants’ accounts either by consulting the health‐care professionals involved or by obtaining first‐hand observational data of similar encounters.

In simple terms, the implications of our findings echo those of other studies, in highlighting the importance for patient of involvement of communication and collaboration between patients and health‐care professionals.^55^, ^56^, ^57^ Our findings further suggest that mindful organizing could be a useful concept for understanding how patients can aid collective comprehension of patient safety risks. We have suggested factors that relate to patients’ engagement in mindful organizing, and which therefore would be usefully developed amongst patients in their interactions with health‐care professionals.

## AUTHORS’ CONTRIBUTIONS

AJA, DMA, DLP and SG conceived and designed the study. SG, DLP, PJL, KM, MJ and NS conducted the focus groups. SG, DLP, PJL, KM and NS were involved in the analysis of the focus groups. All authors contributed to interpretation of the findings and to draft versions of the manuscript. All authors read and approved the final manuscript.
